# Sputum production and salivary microbiome in COVID-19 patients reveals oral-lung axis

**DOI:** 10.1371/journal.pone.0300408

**Published:** 2024-07-25

**Authors:** Korina Yun-Fan Lu, Hend Alqaderi, Saadoun Bin Hasan, Hesham Alhazmi, Mohammad Alghounaim, Sriraman Devarajan, Marcelo Freire, Khaled Altabtbaei

**Affiliations:** 1 Harvard School of Dental Medicine, Boston, Massachusetts, United States of America; 2 Tufts University School of Dental Medicine, Boston, Massachusetts, United States of America; 3 Dasman Diabetes Institute, Dasman, Kuwait; 4 Kuwait Ministry of Health, Sulaibikhat, Kuwait; 5 Department of Preventive Dentistry, Division of Pediatric Dentistry, Umm Al-Qura University, Mekkah, Saudi Arabia; 6 Department of Genomic Medicine and Infectious Diseases, J. Craig Venter Institute, La Jolla, California, United States of America; 7 Department of Medicine, Division of Infectious Diseases and Global Public Health, University of California San Diego, La Jolla, California, United States of America; 8 School of Dentistry, University of Alberta, Edmonton, AB, Canada; Yerevan State Medical University Named after Mkhitar Heratsi, ARMENIA

## Abstract

SARS-CoV-2, a severe respiratory disease primarily targeting the lungs, was the leading cause of death worldwide during the pandemic. Understanding the interplay between the oral microbiome and inflammatory cytokines during acute infection is crucial for elucidating host immune responses. This study aimed to explore the relationship between the oral microbiome and cytokines in COVID-19 patients, particularly those with and without sputum production. Saliva and blood samples from 50 COVID-19 patients were subjected to 16S ribosomal RNA gene sequencing for oral microbiome analysis, and 65 saliva and serum cytokines were assessed using Luminex multiplex analysis. The Mann-Whitney test was used to compare cytokine levels between individuals with and without sputum production. Logistic regression machine learning models were employed to evaluate the predictive capability of oral microbiome, salivary, and blood biomarkers for sputum production. Significant differences were observed in the membership (Jaccard dissimilarity: p = 0.016) and abundance (PhILR dissimilarity: p = 0.048; metagenomeSeq) of salivary microbial communities between patients with and without sputum production. Seven bacterial genera, including *Prevotella*, *Streptococcus*, *Actinomyces*, *Atopobium*, *Filifactor*, *Leptotrichia*, and *Selenomonas*, were more prevalent in patients with sputum production (p<0.05, Fisher’s exact test). Nine genera, including *Prevotella*, *Megasphaera*, *Stomatobaculum*, *Selenomonas*, *Leptotrichia*, *Veillonella*, *Actinomyces*, *Atopobium*, and *Corynebacteria*, were significantly more abundant in the sputum-producing group, while *Lachnoanaerobaculum* was more prevalent in the non-sputum-producing group (p<0.05, ANCOM-BC). Positive correlations were found between salivary IFN-gamma and Eotaxin2/CCL24 with sputum production, while negative correlations were noted with serum MCP3/CCL7, MIG/CXCL9, IL1 beta, and SCF (p<0.05, Mann-Whitney test). The machine learning model using only oral bacteria input outperformed the model that included all data: blood and saliva biomarkers, as well as clinical and demographic variables, in predicting sputum production in COVID-19 subjects. The performance metrics were as follows, comparing the model with only bacteria input versus the model with all input variables: precision (95% vs. 75%), recall (100% vs. 50%), F1-score (98% vs. 60%), and accuracy (82% vs. 66%).

## Introduction

COVID-19, an abbreviation for "coronavirus disease 2019," originates from the SARS-CoV-2 virus and has exerted a global impact affecting millions [1]. While the initial wave of the pandemic may have receded, the persistence of new variants and the ensuing challenges underscores the imperative for sustained investigation into this novel pathogen. It is of vital importance to comprehend its multifaceted effects and develop innovative strategies to ameliorate the severity of clinical manifestations.

Host-specific characteristics exert a pivotal influence on the trajectory of SARS-CoV-2 infection, governing both its progression and severe outcomes [2]. The intricate interplay of local and systemic immune responses assumes a central role in shaping the host’s response to viral infection [2]. Predominant among the clinical expressions of COVID-19 are respiratory symptoms, encompassing cough, chest pain, dyspnea, and sputum production [1]. The viral route of entry, commencing through the oral cavity, followed by infection of respiratory epithelial cells and ensuing pulmonary inflammation, underscores the need to unravel the determinants governing these respiratory symptoms [3]. This necessitates a comprehensive exploration of local factors that explain immune-host responses such as the oral microbiome and inflammatory cytokines, which exert potential influence on symptom severity [3]. While existing literature highlights the connection between cytokine storms and severe manifestations [3], there exists a gap in terms of identifying specific microbiome and cytokine profiles linked to distinct COVID-19 symptoms. The present study endeavors to bridge this gap by directing its focus towards the oral microbiome and cytokine patterns relevant to sputum production—a significant clinical symptom in COVID-19.

Emerging research revealed a bidirectional interaction between COVID-19 and the oral microbiome [4]. Investigations have revealed that the oral immune system response may be influenced by the oral microbiome [4]. It was shown that patients infected with the virus exhibit distinctive oral microbial compositions compared to their non-infected counterparts [4]. This signifies the impact of the oral microbiota on the immune response during the acute phase of infection, thereby potentially contributing to the exacerbation of the clinical manifestation of COVID-19.

This study aimed to comprehensively elucidate the intricate interplay between the oral microbiome and cytokine profiles, especially those associated with respiratory symptoms, which is an area that remains incompletely explored. In this context, the present research aims to cover this gap. A primary objective involves the comparative analysis of oral microbiome compositions and cytokine profiles among individuals presenting with specific respiratory symptoms, with a particular emphasis on sputum production.

## Materials and methods

This study was a collaborative joint study between the Harvard School of Dental Medicine (HSDM), J Craig Venter Institute (JCVI), the Ministry of Health in Kuwait, and the University of Alberta. The study was approved by JCVI, Harvard, the Kuwait Ministry of Health, and the University of Alberta. Informed consent was obtained from all enrolled participants (Kuwait Ministry of health ethics approval: #2020/1462 Harvard: IRB21-1492, University of Alberta: Pro00125245, JVCI: exempt due to secondary analysis of de-identified samples).

### Study design

A convenient sample strategy was used to recruit patients admitted at multiple Covid-19 centers in Kuwait between July 24th and September 4th, 2020. The data collection took place at three hospitals in Kuwait: Al Farwaniya Hospital, Jaber Al Ahmed Hospital, and Kuwait Field Hospital. Data were collected from those who provided positive consent and who tested positive for SARS-CoV-2 by RT-PCR from nasopharyngeal swabs (n = 50). Saliva and serum samples were collected from individuals within 48 hours of PCR-confirmed COVID-19 diagnosis. The basic demographic and clinical information (including medical history, medication, weight, height, waist circumference, neck circumference, blood group, respiratory rate, and oxygen supplementation in liters for those on oxygen) of the study participants was obtained.

The severity of the disease was stratified into mild: hospitalized, no oxygen therapy (n = 11); moderate: hospitalized, low-flow oxygen (<10 L/min) (n = 28); and severe: hospitalized, high-flow oxygen (>10 L/min) (n = 11). Mild and moderate groups were combined under “non-severe” in the present analysis.

### Saliva collection

15 mL plastic centrifuge tubes were prelabeled with the date and subject number. We then marked the 4 mL line of the tube. A parafilm was used to stimulate saliva.

Prior to sample collection, the saliva collection tube was placed in a cup with ice. A nurse, supervised by the research coordinator, would demonstrate how to provide saliva to the patient. Each subject was instructed to take a sip of water and rinse their mouth, swallow the water, chew the piece of parafilm, and then use their tongue to push saliva as it formed into the tube. They were then instructed to place the tube back in the cup with the ice cube while they waited for more saliva to form. The saliva was collected until it reached the line (4 mL) on the tube, considering the liquid region of the saliva sample (not the foamy regions). Once the patient finished providing the saliva sample, they notified the nurse. The nurse tightened the cap on the tube, wiped it with alcohol, placed the tube in the collection rack in the cooler with ice and discarded the other materials.

### Blood collection

Serum samples were collected using standard venipuncture techniques in 7.5 mL BD Vacutainer Serum marbled topped tubes with clot activator for serum. Samples were collected at the hospital.

### Sample processing

The samples were transferred to the Jaber Al Ahmad Hospital laboratory in containers with dry ice. The laboratory technician received the samples and processed them on the same day of sample collection within no more than 3 hours. Saliva samples were centrifuged at 2000 x g for 5 min, and the supernatants were separated from the pellets and transferred into different tubes. Serum samples were allowed to sit upright in racks at room temperature for 30 min prior to centrifuging at 2000 xg for 10 min at room temperature. All the samples were stored at –80°C and were transferred from Kuwait to JCVI. The samples were placed on dry ice during shipment with a monitoring device to ensure that the samples were frozen during the transfer.

### DNA extraction, library preparation, and sequencing

DNA was extracted from samples using Qiagen’s AllPrep Bacterial DNA/RNA/Protein Kit (Cat# 47054; QIAGEN, Hilden, Germany) according to the manufacturer’s instructions. Step 3 was modified to use MP Biomedicals™ FastPrep-24™ Classic Bead Beating Grinder and Lysis System (Cat# MP116004500; FisherScientific) for 1 minute instead of vortexing for 10 minutes. All recommended products were used, and, at step 2, ß-ME was used instead of DTT.

Sequencing was done on the Illumina MiSeq platform. V4 hypervariable region was sequenced using the following forward (MSV4Read1) and reverse primers (MSV34Index1), 5’-TATGGTAATTGTGTGCCAGCMGCCGCGGTAA-3’ and 5’-ATTAGAWACCCBDGTAGTCCGGCTGACTGACT-’3.

Samples were deposited in NIH SRA under the accession number PRJNA948421.

### Cytokine abundance measurements

Serum samples were analyzed using the Immune Monitoring 65-Plex Human ProcartaPlex Panel (Cat# EPX650-16500-901; ThermoFisher Scientific, Vienna, Austria), according to manufacturer’s instructions, and runs on the Luminex 200 system (Luminex Corporation, Austin, Texas, USA). This kit measured immune function by analyzing 65 protein targets in a single well, including cytokine, chemokine, growth factors/regulators, and soluble receptors. The provided standard was diluted 4-fold to generate a standard curve, and high and low controls were also included.

### Amplicon sequence variants (ASVs)

Analysis was performed at the amplicon sequence variant level: DNA sequences containing no sequencing errors after algorithmic correction. The paired-end DADA2 [5] workflow of QIIME2 v2022.2.0 [6] was used for detecting ASVs and quantifying abundance implemented using the amplicon.py module of VEBA [7]. More specifically, this workflow uses the following protocol: 1) qiime tools import of paired-end reads; 2) DADA2 denoising of paired reads and ASV detection by qiime dada2 denoise-paired (forward_trim = 251, reverse_trim = 231, min-overlap = 12); 3) taxonomic classification of ASVs using the precompiled silva-138-99-nb-classifier.qza model (Silva_v1383__SSURef_NR9); and 4) conversion into generalized machine-readable formats (e.g., QIIME2 Artifact and BIOM formatted files → tab-separated value and Fasta-formatted files).

### Statistical analysis

Demographic data were collected without identified information, and descriptive statistics were computed for each variable, including mean and Standard Deviation (SD) for continuous variables, and counts and percentages for categorical variables. All data were accessed on October 9^th^, 2022. The chi-square test was utilized to assess the association between categorical variables, while the t-test was employed to evaluate differences in means between groups for continuous variables.

Sixty-five blood and salivary biomarker data were analyzed. For each biomarker, the median and interquartile range (IQR) were calculated for individuals with no sputum production (n = 27) and those with sputum production (n = 23). The non-parametric Mann-Whitney U test was utilized to assess the significance of differences between the two groups for each biomarker. A p-value threshold of less than 0.05 was considered statistically significant.

The aforementioned analyses were conducted using STATA SE 17.0 (StataCorp, College Station, TX, USA), with a significance level of p < 0.05 established for determining statistical significance.

### Microbiome statistical analysis

Bacterial composition data were analyzed, and subsequent data manipulation and adjustments were carried out using Microsoft Excel. To investigate significant differences in bacterial membership between the Sputum group (n = 23) and the Non-Sputum (control) group (n = 27), the following approach was taken. For each bacterial species, the presence of bacteria was represented as either a "1" (present) or a "0" (absent) for each subject. The number of "0s" (no bacteria) was then counted for each bacterial species in both groups. To assess the significance of differences in bacterial membership, a chi-square test was conducted for each bacterial species. The chi-square test determined whether the distribution of "0s" (absence of bacteria) significantly differed between the two groups. After obtaining chi-square test results from STATA, the subsequent data processing, adjustment of p-values using methods such as the Benjamin-Hochberg procedure, and table formatting were performed using Microsoft Excel. Adjusted p-values were calculated in Excel to control for multiple testing, and the final table was generated to present the adjusted p-values along with additional relevant information, including the number of subjects in each group with the bacteria. Compositionally-aware differential abundance analysis was carried out using the ANCOM-BC [8] implementation in DATest package [9]. Beta diversity using PhILR [9] and beta-diversity was done on the automated pipeline FALAPhyl (available http://github.com/khalidtab/falaphyl).

### Machine learning logistic regression

Two logistic regression machine learning models were employed to evaluate the predictive capability of oral microbiome, salivary, and blood biomarkers in predicting sputum production symptoms in COVID-19 subjects. The output variable was binary, indicating the presence or absence of sputum symptoms. For Model 1, features from oral microbiome data, blood and salivary biomarkers, and clinical and demographic variables were included. Model 2 utilized only the microbiome input variables. The objective was to determine which model performs better in predicting sputum production: the model with the entire dataset (oral microbiome, blood and salivary biomarkers, clinical and demographic variables) or the model with only the oral microbiome data.

Categorical variables were transformed into binary dummy variables using one-hot encoding, allowing their use in machine learning models. Examples of these variables included sex, medical history, and periodontal status. Continuous variables in this dataset had different scales, which could potentially introduce bias into the machine learning algorithm by giving higher weight to features with larger magnitudes. To address this issue, feature scaling was performed using the Standard Scaler method, which centers and scales the dataset using the Z-score.

The dataset was divided into a training set (80%) for model development and a test set (20%) for assessing predictive performance. Due to the small sample size, bootstrapping and 5-fold cross-validation techniques were employed to enhance the validity of the models. Model performance was evaluated using recall, precision, F1, and accuracy, calculated from the confusion matrix. Recall measured the model’s ability to correctly identify positive instances, precision measured the accuracy of the positive predictions made, and accuracy provided the overall correctness of the model.

To identify the most influential features in predicting sputum production symptoms in COVID-19 subjects, a feature importance analysis was conducted for the best-performing model. Python was used for the machine learning analysis.

## Results

### Demographics

A total of 50 individuals who tested positive for SARS-CoV-2 were included in the study, consisting of 23 individuals with sputum production and 27 individuals without sputum production (Table 1). The average age for individuals with sputum production was 47.3, which was comparable to the average age of individuals without sputum production. The severity of the disease was categorized as mild (hospitalized without oxygen therapy, n = 11), moderate (hospitalized with low-flow oxygen <10 L/min, n = 28), and severe (hospitalized with high-flow oxygen >10 L/min, n = 11). The analysis combined the mild and moderate groups into a "non-severe" category: 82.6% of the participants with sputum production exhibited non-severe symptoms and 17.4% exhibited severe symptoms. Among the subjects exhibited sputum production, 56.5% were males, and 43.5% were females. Diabetes was present in 47.8% of the sputum positive group and 40.7% in the sputum-negative group. Heart disease was observed in 8.7% of the sputum-positive group and 22.2% of the sputum-negative group. 82.6% of those with sputum production experienced shortness of breath, which was significantly higher than the group without sputum production (48.1%). Chest pain was higher in those with sputum production, with 30.4% in the sputum-positive group and 18.5% in the sputum-negative group, but not to a statistically significant level. Asthma and emphysema were comparable between individuals with and without sputum production.

**Table 1:** Demographics and medical information of the study population.

Variables		Sputum (n = 23)		No sputum (n = 27)		Total (n = 50)	P value
**Age, years, mean (SD)**		47.3 (12.0)		53.8 (14.5)		50.8 (13.7)	0.1
**Sex**	Female	10	43.5%	12	44.4%	22 (44%)	0.9
	Male	13	56.5%	15	55.6%	28 (56%)	
**COVID-19 severity**	Non-severe	19	82.6%	20	74.1%	39 (78%)	0.5
	Severe	4	17.4%	7	26.9%	11 (22%)	
**Diabetes**	Yes	11	47.8%	11	40.7%	22 (44%)	0.6
	No	12	52.2%	16	59.3%	28 (56%)	
**Heart disease**	Yes	2	8.7%	6	22.2%	8 (16%)	0.2
	No	21	91.3%	21	77.8%	42 (84%)	
**SOB**	Yes	19	82.6%	13	48.1%	32 (64%)	0.01*
	No	4	17.4%	14	51.9%	18 (36%)	
**Smoking**	Yes	1	4.3%	0	0%	1 (2%)	0.3
	No	22	95.7%	27	100%	49 (98%)	
**Chest Pain**	Yes	7	30.4%	5	18.5%	12 (24%)	0.3
	No	16	69.6%	22	81.5%	38 (76%)	
**Asthma**	Yes	4	17.4%	1	3.7%	5 (10%)	0.1
	No	19	82.6%	26	96.3%	45 (90%)	
**Emphysema**	Yes	0	0%	1	3.7%	1 (2%)	0.4
	No	23	100%	26	96.3%	49 (98%)	
**WBC**		5.8 (1.5)		6.8 (3.7)		6.3 (2.9)	0.3

### Oral microbiome

**Fig 1:**
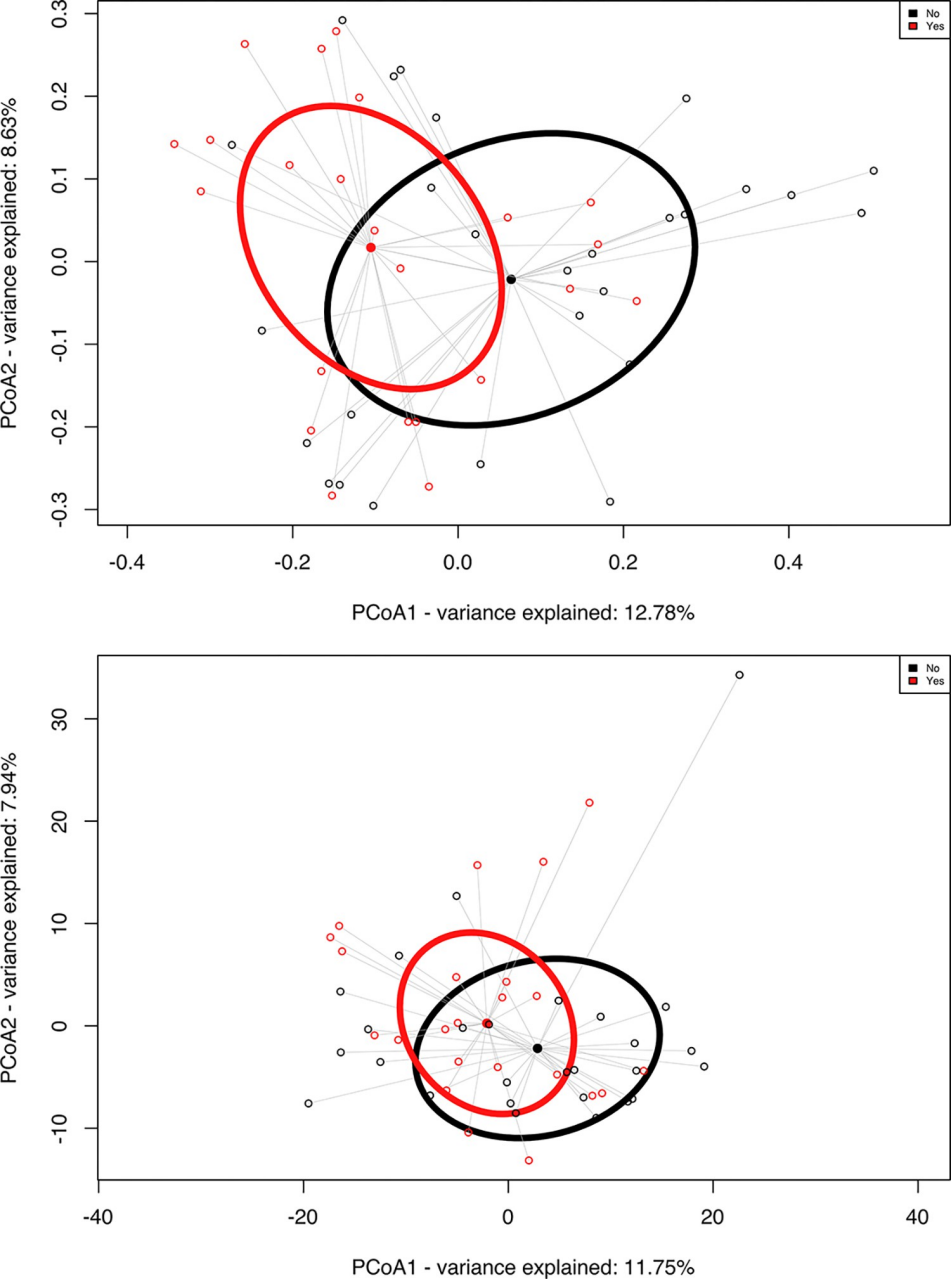
Analysis of salivary microbiome between the 2 groups, sputum positive (red, n = 23) and sputum-negative (black, n = 27). A:​​Principal coordinates analysis (PCoA) of Jaccard dissimilarity matrices between COVID-19 positive patients with sputum production and without sputum production. Ellipsoids show the deviation of spread of each group (ADONIS, p = 0.016). B: PCoA of Euclidean distances of PhILR distances, demonstrating the three clusters between the conditions (ADONIS, p = 0.048).

**Fig 2:**
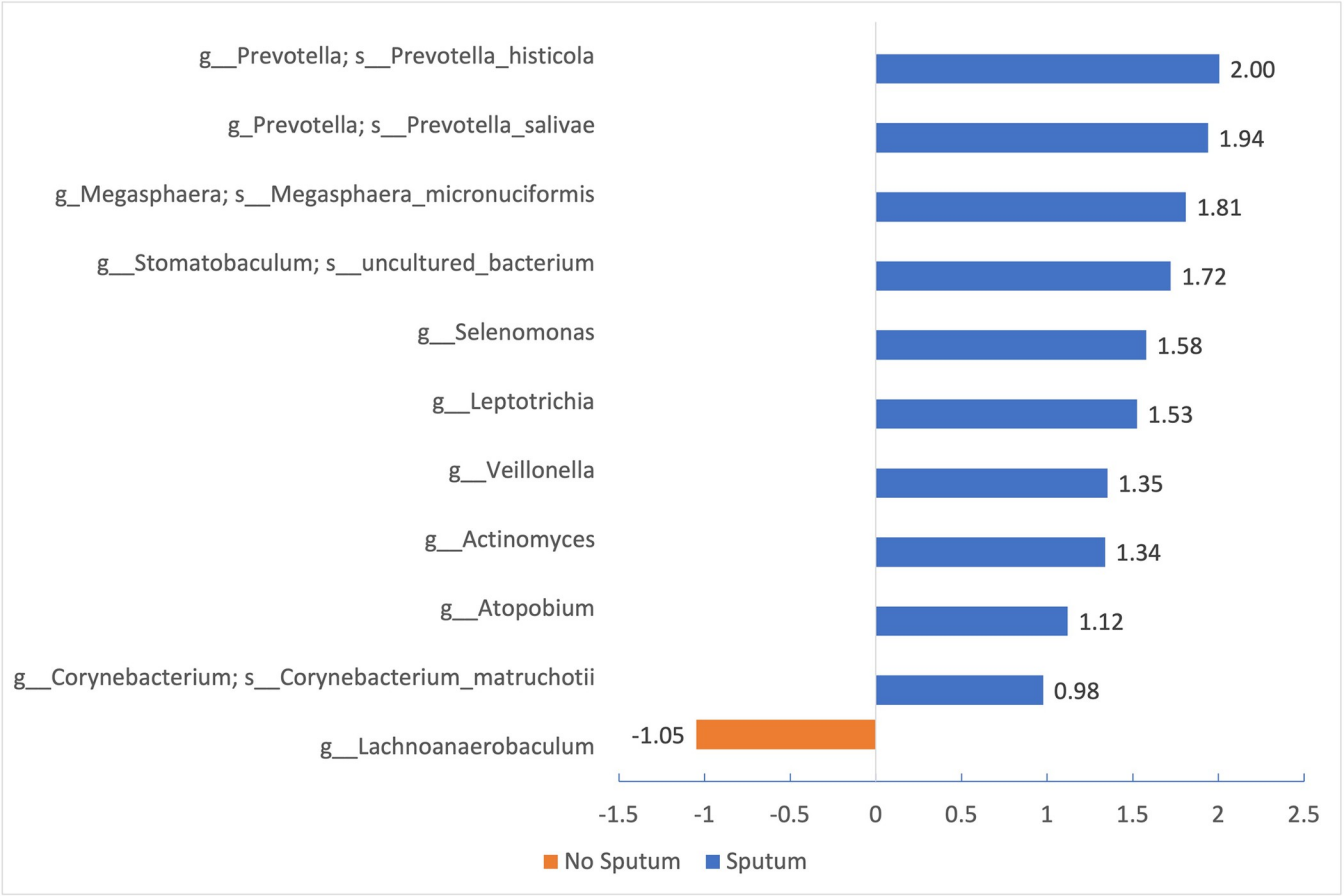
Differentially abundant bacteria (P<0.05, ANCOM-BC) in participants with Sputum production (blue) and without sputum production (orange).

Our study showed significant differences in the membership (Jaccard dissimilarity: p = 0.016) and abundance (PhILR dissimilarity: p = 0.048; metagenomeSeq) of salivary microbial communities between COVID-19 patients with and without sputum production (Fig 1 and Table 2). In terms of membership, we found that seven bacterial genera were present in statistically higher proportions of patients with sputum production (p<0.05, Fisher’s exact test). These bacteria were: *Prevotella*, *Streptococcus*, *Actinomyces*, *Atopobium*, *Filifactor*, *Leptotrichia*, and *Selenomonas* (Table 2). For abundance, nine bacterial genera were presented in significantly higher abundance in the group with sputum production, including *Prevotella*, *Megasphaera*, *Stomatobaculum*, *Selenomonas*, *Leptotrichia*, *Veillonella*, *Actinomyces*, *Atopobium*, and *Corynebacteria*, and one genus, *Lachnoanaerobaculum*, was significantly more abundant in the group without sputum production (p<0.05, ANCOM-BC) (Fig 2).

**Table 2:** Bacteria that are significantly different in their membership between Sputum group and control group.

Bacteria genera	Adjusted p value (Benjamin-Hochberg)	number of subjects with this bacterium (Control group; n = 27)	number of subjects with this bacterium (Sputum group; n = 23)
*Prevotella*	0.04	1 (3.7%)	5 (21.7%)
*Filifactor*	0.03	1 (3.7%)	9 (39.1%)
*Actinomyces*	0.03	8 (3.0%)	13 (56.5%)
*Atopobium*	0.03	19 (70.4%)	21 (91.3%)
*Leptotrichia*	0.04	12 (44.4%)	18 (78.3%)
*Selenomonas*	0.04	12 (44.4%)	18 (78.3%)
*Streptococcus*	0.04	6 (22.2%)	12 (52.2%)

All are present with a significantly higher proportion in patients with sputum production.

### Cytokines

We profiled saliva and serum samples to characterize oral mucosal and systemic responses following SARS-CoV-2 infection more comprehensively. Out of the 65 serum and salivary cytokines, five serum and one salivary cytokine were significantly different between individuals with and without sputum production. The serum Eotaxin2/CCL24 (chemokine ligand 24) and salivary IFN gamma (Interferon‐gamma) were significantly elevated in individuals who reported sputum production, and MCP3/CCL7 (monocyte-chemotactic protein 3/Chemokine ligand 7), MIG/CXCL9 (Monokine induced by gamma/Chemokine ligand 9), IL1 beta (interleukin 1 beta), and SCF (stem cell factor) were found to be significantly lower in individuals who reported sputum production during the acute phase of COVID-19 infection (Table 3). It is important to note that the measured levels included both chemokines and cytokines. Specifically, Eotaxin2/CCL24, MCP3/CCL7, and MIG/CXCL9 are chemokines, while IFN gamma, IL1 beta, and SCF are cytokines.

**Table 3:** Blood biomarkers and salivary biomarkers that are significantly different between individuals with and without sputum production.

		No sputum (n = 27), median (IQR)	Sputum (n = 23), median (IQR)	P value
**Blood Biomarkers**	Eotaxin2/CCL24	711 (282, 1603.5)	1325 (601, 1538)	0.047
	MCP3/CCL7	11 (6,19)	8 (6,11)	0.042
	MIG/CXCL9	28.5 (1, 92)	8.5 (-5, 43)	0.011
	IL1 beta	-4 (-6,1)	-6 (-7,-5)	0.011
	SCF	34 (14, 131)	14 (5,29)	0.047
**Salivary Biomarkers**	IFNgamma	0.688 (0.293, 1.111)	1.245 (0.234, 2.813)	0.047

IQR: interquartile range.

### Machine learning (ML) logistic regression models

Table 4 compares the performance of the Model 1 and 2. Model 1 includes features from oral microbiome data, blood and salivary biomarkers, and clinical and demographic variables, while Model 2 utilized only the microbiome input variables. Model 2 achieved higher precision (0.95 vs. 0.75), recall (1.00 vs. 0.50), F1-score (0.98 vs. 0.60), and accuracy (0.82 vs. 0.66).

**Table 4:** Comparison of performance of two logistic models.

Metric	Model 1 (All data including oral microbiome, blood and salivary biomarkers, clinical and demographic variables)	Model 2 (Only Bacteria)
**Precision**	75%	95%
**Recall**	50%	100%
**F1-Score**	60%	98%
**Accuracy**	66%	82%

**Fig 3:**
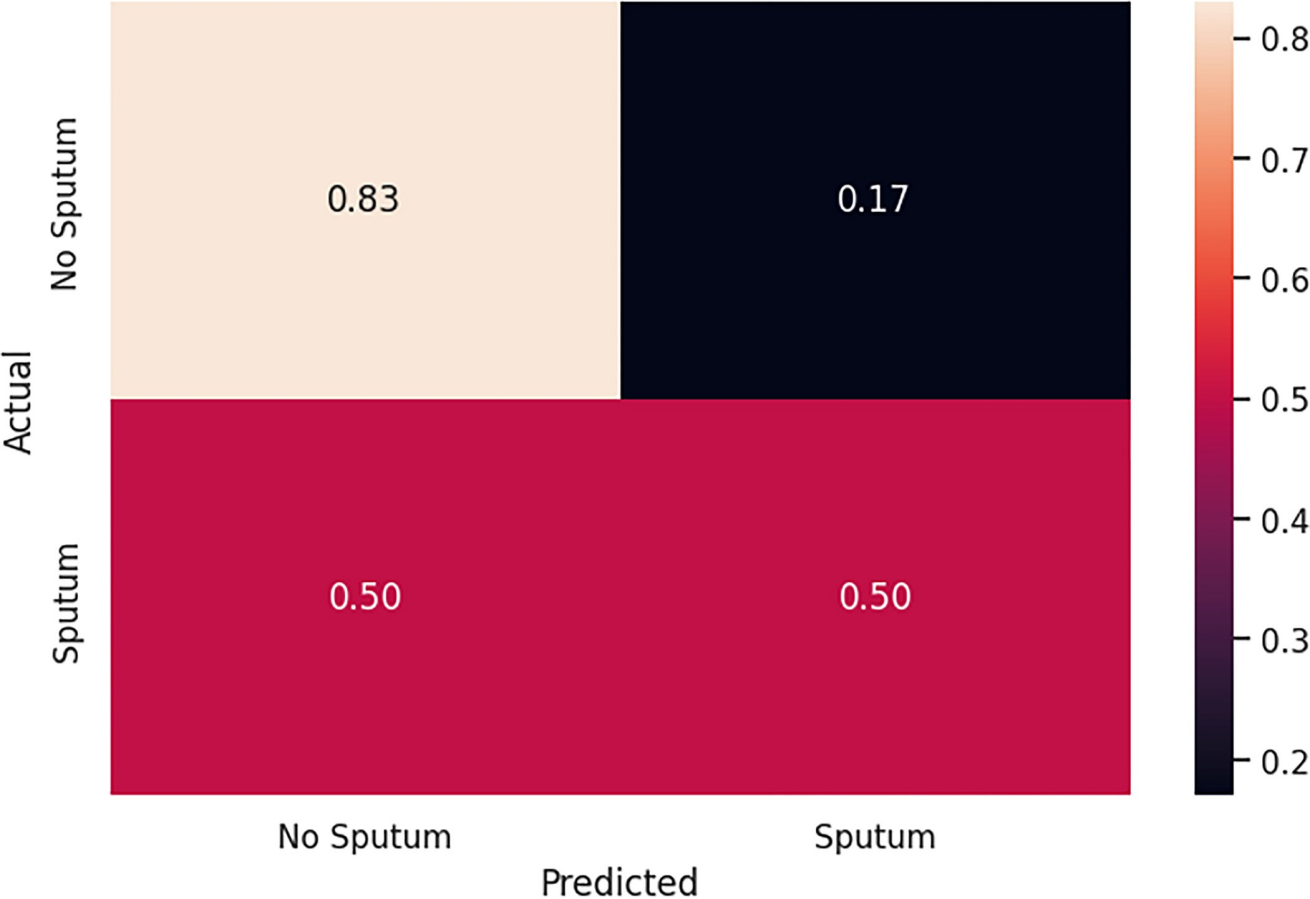
Confusion matrix of Model 1 with bacteria and biomarkers.

**Fig 4:**
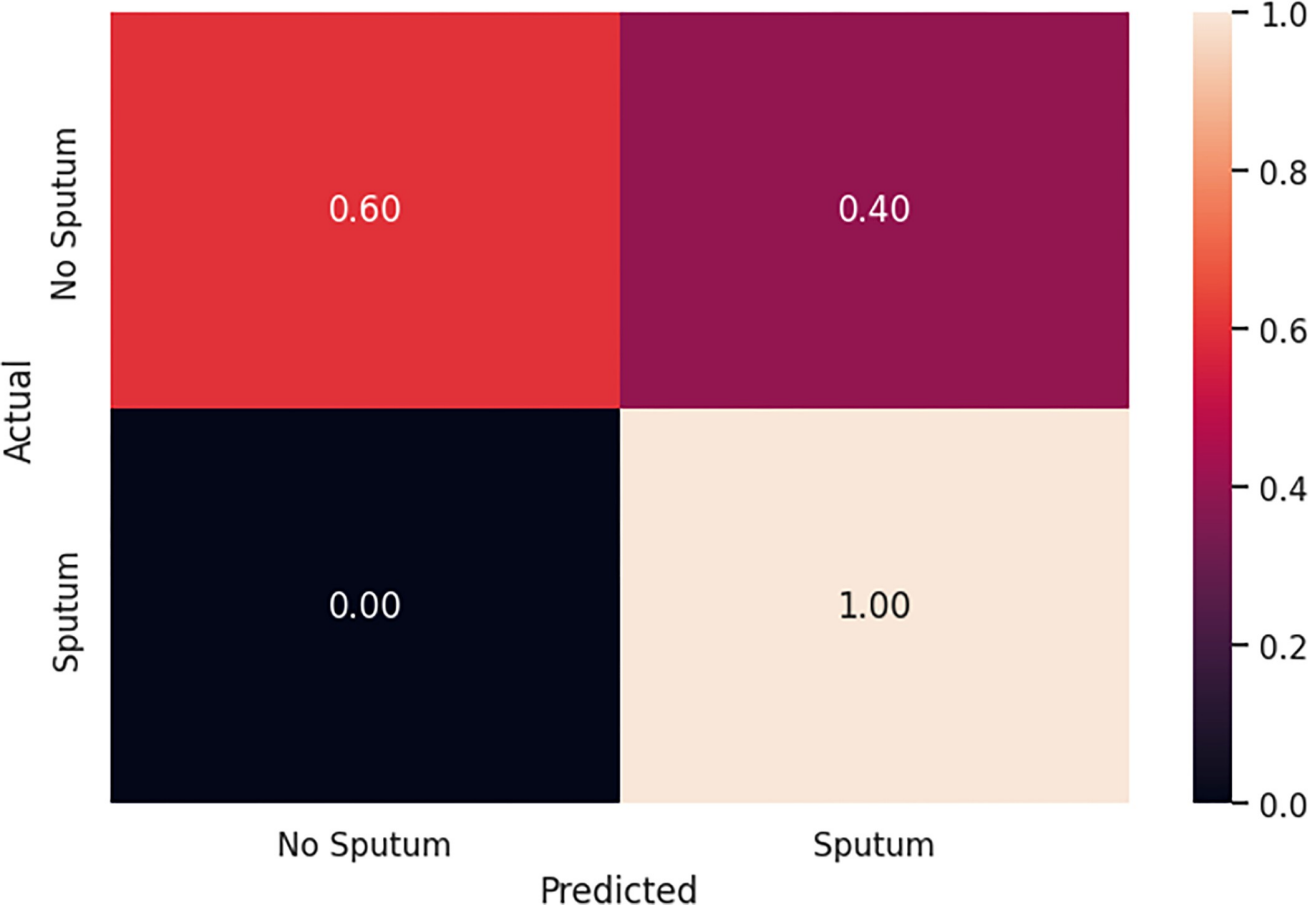
Confusion matrix of Model 2 with only the bacteria.

The performance of the two logistic regression models was compared using confusion matrices. For Model 1, the true positives (TP) and true negatives (TN) were 0.50 and 0.83, respectively (Fig 3). The model exhibited false positives (FP) at 0.17 and false negatives (FN) at 0.50. In comparison, Model 2 demonstrated improved performance (Fig 4). It achieved TP of 1.00 and TN of 0.60. Although the model showed a higher rate of FP at 0.40, it had no FN.

**Fig 5:**
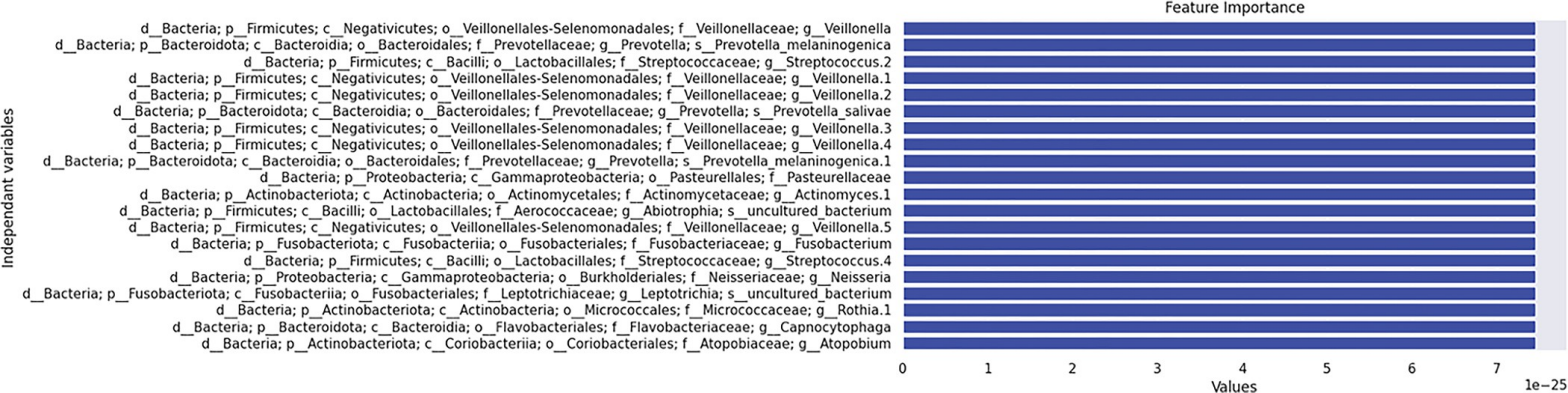
Feature importance demonstrating the most important features that predicted sputum production for Model 2 (bacteria only).

The feature importance graph (Fig 5) illustrates the most important features that predicted sputum production for the best-performing model, namely Model 2 with only bacteria. Notably, genera such as *Veillonella*, *Prevotella*, and *Streptococcus* appear prominently, indicating their strong association with sputum production symptoms.

**Fig 6:**
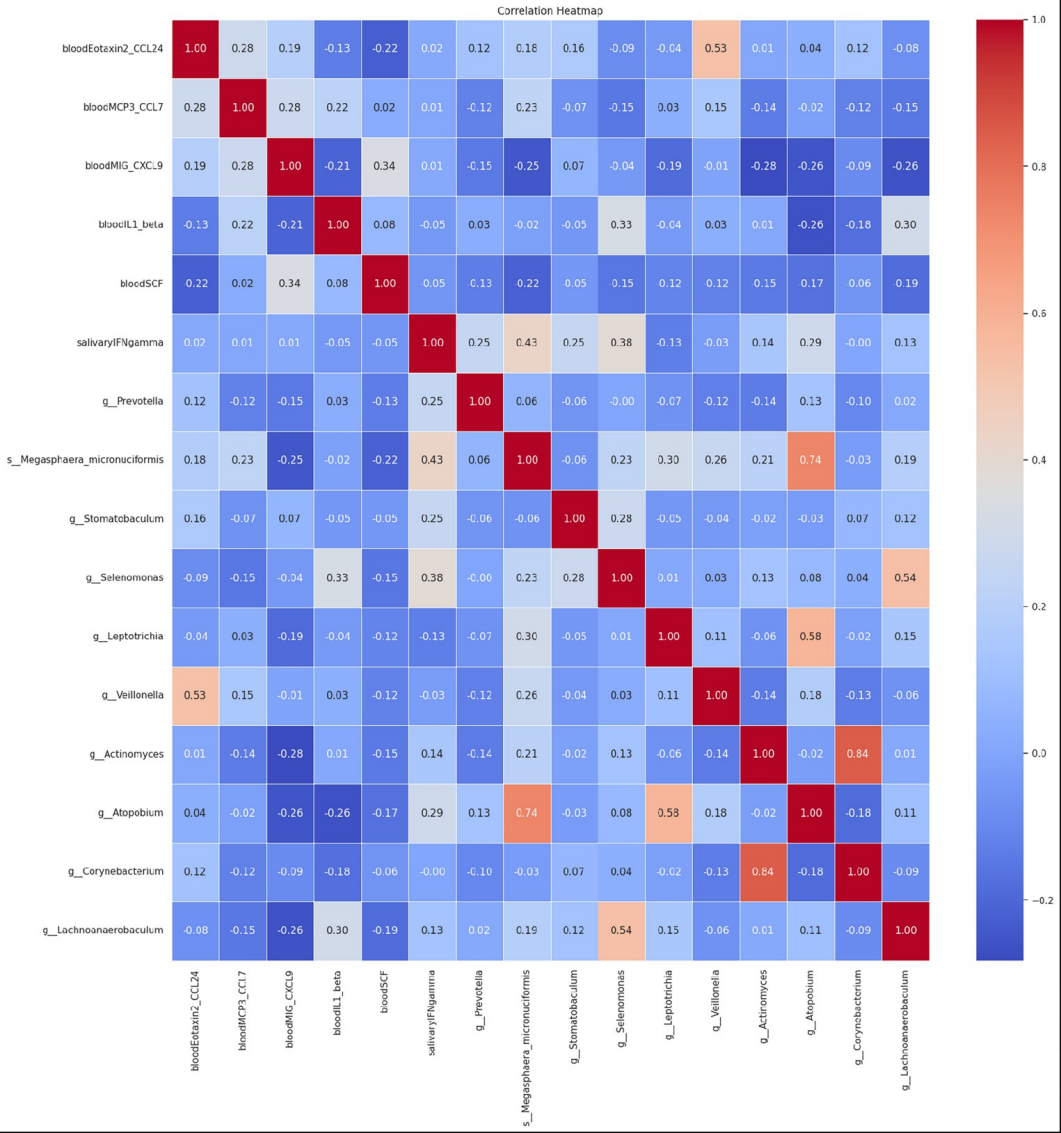
Correlation heatmap between bacteria genera and biomarkers that are significantly different in their abundance between COVID-19 patients with or without sputum production.

The correlation heatmap (Fig 6) provides a visual representation of the relationships between various biomarkers and bacterial genera that are statistically different between COVID-19 patients with or without sputum production. A strong positive correlation (r = 0.53) was observed between the blood biomarker Eotaxin2 (CCL24) and the bacterial genus *Veillonella*. Additionally, a significant positive correlation (r = 0.74) was noted between the bacterial genera *Atopobium* and *Megasphaera micronuciformis*, suggesting a potential co-occurrence or interaction between these taxa. Conversely, a negative correlation (r = -0.28) was identified between the blood biomarker IL-1 beta and the bacterial genus *Stomatobaculum*. Other notable correlations include a positive relationship (r = 0.58) between *Atopobium* and *Leptotrichia*, and a negative correlation (r = -0.26) between CCL7 and *Veillonella*.

## Discussion

### Oral microbiome

This study demonstrated a distinct bacterial cluster between people who developed sputum production compared to those who did not exhibit sputum symptoms during the acute phase of SARS-Cov-2 infection. Results showed distinct oral microbiome membership and abundance between patients with sputum production and controls. Seven bacteria genera were present in higher proportions of patients who exhibited sputum production: *Prevotella*, *Filifactor*, *Actinomyces*, *Atopobium*, *Leptotrichia*, *Selenomonas*, and *Streptococcus* (Table 2). Additionally, there was a major difference in the abundance of bacteria between those who manifested sputum production compared to those who didn’t. Nine bacterial genera exhibited significantly higher levels in the group with sputum production. These included *Prevotella*, *Megasphaera*, *Stomatobaculum*, *Selenomonas*, *Leptotrichia*, *Veillonella*, *Actinomyces*, *Atopobium*, and *Corynebacteria*. Conversely, one genus, *Lachnoanaerobaculum*, was notably more abundant in the group without sputum production (Fig 2). Notably, *Prevotella*, *Actinomyces*, *Atopobium*, *Selenomonas*, and *Leptotrichia* displayed differences both in their membership and abundance.

The identification of distinct bacterial clusters and differences in oral microbiome membership and abundance between COVID-19 patients with and without sputum production has significant implications, particularly when considering the potential connections between these findings and oral health conditions, particularly periodontitis. *Prevotella*, *Atopobium*, *Filifactor*, and *Actinomyces*, for instance, have well-established connections with periodontal health and oral infections [10–12]; *Leptotrichia* species can exhibit invasive traits, potentially causing various infections, and *Megasphaera* species are linked to periodontal disease and polymicrobial infections [13, 14]. The association of these bacteria to periodontal diseases is of significance as periodontitis has been shown to be associated with an increased likelihood of developing COVID-19 symptoms [15, 16]. Individuals with underlying periodontal issues might face an increased susceptibility to COVID-19 complications due to oral-systemic links [16]. Emerging research has revealed a potential association between moderate-to-severe periodontitis and heightened risks of COVID-19 complications, including severe outcomes such as death, ICU admission, and the need for assisted ventilation [15]. These findings suggest that periodontal disease may exacerbate the severity of COVID-19 infections, possibly due to shared inflammatory pathways and risk factors with chronic inflammatory conditions known to influence COVID-19 outcomes [15]. Although we didn’t perform oral exams on the individuals in our study, it is essential to consider the potential impact of periodontal health on respiratory symptoms like sputum production, as it has been associated with an increased likelihood of COVID-19 symptoms [15].

We propose two potential pathways that periodontitis could be linked to COVID-19 respiratory symptoms such as sputum production: periodontitis is recognized as a chronic inflammatory condition, and this inflammation could potentially exacerbate the severity of COVID-19 symptoms [15]. Secondly, periodontal pathogens, including specific bacteria like *Prevotella*, *Atopobium*, *Filifactor*, *Actinomyces*, *Leptotrichia*, and *Streptococci*, may have the capacity to migrate into the respiratory tract during a COVID-19 infection, causing respiratory symptoms like sputum production [10, 11, 13, 17].

The temporal origin of these bacteria identified in sputum, whether existing prior to COVID-19 infection and exacerbating symptoms such as sputum production, or emerging during the infection’s progression, remains unclear. Previous studies documented that in the sputum samples of healthy subjects, *Firmicutes*, *Bacteroidetes* and *Actinobacteria* were the major phyla, and *Streptococcus*, *Veillonella*, *Prevotella*, *Haemophilus*, *Actinomyces* and *Rothia* were among the dominant genera [18,19]. Therefore, a hypothesis can be formulated, suggesting that the oral disease-related bacterial species we identified in individuals with sputum production might have already been present prior to COVID-19 infections due to specific periodontal diseases or dental caries. These bacterial species could potentially contribute to exacerbating respiratory symptoms and intensifying inflammation. This underscores the significant role of oral health as a potential gateway to systemic diseases. Notably, a previous analysis of the same dataset unveiled marked differences in oral microbiome profiles between individuals with severe symptoms and those with mild symptoms [20]. This suggests the potential impact of the oral immune system and microbiome on the severity of COVID-19 symptoms.

### Cytokines and chemokines

Our analysis demonstrated that certain cytokines were associated with sputum production. Out of the 65 serum and salivary cytokines we analyzed, serum Eotaxin2/CCL24, and salivary IFN-gamma were elevated in individuals who reported sputum production, and MCP3/CCL7, MIG/CXCL9, IL1 beta, and SCF were found to be lower in individuals who reported sputum production during the acute phase of COVID-19 infection.

These findings are particularly intriguing when considering the role of cytokines and chemokines in immune responses in the context of COVID-19 and oral dysbiosis. Strong evidence showed that the oral microbiome plays a crucial role in the development and maintenance of the immune system’s homeostasis [21]. Oral dysbiosis, characterized by an imbalance or perturbation in the composition of the oral microbiome, profoundly influences the host immune response and the intricate network of cytokines involved [22]. When the oral microbiome experiences dysregulation, often associated with conditions like periodontal disease, the immune system mounts a multifaceted response [23]. This immune reaction involves the secretion of various cytokines, which are typically upregulated in response to oral dysbiosis, driving inflammation to combat the overgrowth of pathogenic bacteria [23]. Many proinflammatory cytokines, including IFN-gamma [24,25] and Eotaxin2/CCL24 [26] which are elevated in our study’s group with sputum production (Table 3), have been shown to be correlated with worse clinical outcomes associated with COVID-19. Specifically, the elevated salivary level of IFN-gamma indicated the onset of inflammation right at the virus’s entry point. As previously proposed, the respiratory symptoms of COVID-19 could be intercorrelated with the complex interplay between periodontal diseases, oral dysbiosis, and cytokine dysregulation [27]. The heightened levels of these two cytokines in individuals with sputum production may suggest an overly robust immune response in response to virus and dysbiosis, potentially exacerbating symptom severity. Moreover, cytokines MCP3/CCL7, MIG/CXCL9, IL1 beta, and SCF, key in viral infection immune responses, exhibited reduced levels in COVID-19 patients with sputum production during the acute phase in our study. This suggests potentially less effective immune defense against the virus. MCP3/CCL7 draws monocytes, T cells, and dendritic cells to infection sites [28], while MIG/CXCL9 attracts T cells and natural killer cells [29]. Pro-inflammatory cytokine IL-1 beta initiates immune responses [30], and SCF supports white blood cell, including T cell, production [31]. Lower cytokine levels might imply a compromised immune reaction, potentially resulting in severe infections or extended recovery.

In terms of limitations, this study employed a cross-sectional design, and the relatively modest sample size could potentially limit our capacity to detect significant differences among different presentations of respiratory symptoms. Additionally, due to pandemic-related circumstances, we were unable to gather data on periodontal health and dental caries, despite the potential influence of oral health status on the salivary microbiome. It’s important to acknowledge the limitations inherent in our study, emphasizing the necessity for subsequent research endeavors. We also acknowledge that any definitive connection between these taxa and sputum production should be verified with actual sputum samples from within the respiratory cavity.

### Machine learning (ML) analysis predicting sputum production

The results of our logistic regression ML models indicate that the model incorporating only bacterial data outperformed the model that included a combination of oral microbiome, blood and salivary biomarkers, and clinical and demographic variables. Specifically, the model with only bacterial input achieved higher precision (0.95 vs. 0.75), recall (1.00 vs. 0.50), F1-score (0.98 vs. 0.60), and accuracy (0.82 vs. 0.66). This suggests that bacterial composition alone may be a more robust predictor of sputum production in COVID-19 patients than a broader array of biomarkers and clinical data. These findings highlight the significant role of the oral microbiome in respiratory health and suggest that bacterial profiling could be a key factor in developing predictive models for COVID-19 symptoms. Furthermore, the feature importance analysis corroborates these results, with key bacterial genera like *Veillonella* and *Prevotella* emerging as critical predictors, aligning with other studies that have identified these taxa as influential in respiratory conditions. This emphasizes the potential of microbiome-focused diagnostics and therapeutics in managing and predicting respiratory symptoms in COVID-19.

## Conclusion

There was a notable distinction in the oral bacterial composition and abundance, alongside levels of inflammatory cytokines in both serum and saliva, between patients exhibiting sputum production and those without it. These variations suggest distinct host immune responses between the two groups. Particularly noteworthy is the heightened presence and abundance of numerous pathogenic bacteria associated with oral diseases in individuals experiencing sputum production. Furthermore, the machine learning model indicates that bacterial composition alone can be a robust predictor of sputum production in COVID-19 patients. This emphasizes the critical role of the oral microbiome in respiratory health and suggests its potential in developing predictive models for COVID-19 symptoms. Considering the oral cavity as the initial point of entry for COVID-19, an intriguing hypothesis emerges: compromised oral health could potentially act as a contributing factor, enhancing the likelihood of respiratory symptoms linked to SARS-CoV-2, notably including the production of sputum.
